# Supplementary material for: Augmented Antitumor Effect of Unripe Rubus coreanus Miquel Combined with Oxaliplatin in a Humanized PD-1/PD-L1 Knock-In Colorectal Cancer Mouse Model
Source: Cells. 2022 Sep 14;11(18):2876. doi: 10.3390/cells11182876 (PMC9496898; doi:10.3390/cells11182876)
Supplement: Supplementary file 1 [file cells-11-02876-s001.zip › Figure S1.pdf]

Supplementary materials

Combination index (CI) of RCE plus Oxa

The combination of RCE and Oxa was quantitatively determined using the Chou–Talalay method for drug combinations [1,2]. The combination index (CI) values were based on the drug–drug interactions and effect equation and calculated using the formula CI = (D_1/D_{X1}) + (D_2/D_{X2}), where D_1 and D_2 represent the doses of drugs 1 and 2 when the combination reached a certain cell viability inhibitory rate and D_{X1} and D_{X2} represent the doses of single drugs 1 and 2 under the cell viability inhibitory rate. A CI value <1 indicates synergism, CI = 1 indicates addictive interaction, and CI >1 indicates antagonism. In our study, CI values were determined for each concentration of RCE and Oxa and their combination in the co-culture cell model with hPD-L1 MC38 cells and hPD-1 tumor-infiltrating $CD3^{+}CD8^{+}$ T cells for 72 h.

Supplementary References

1. Chou, T.C.; Talalay, P. Quantitative analysis of dose-effect relationships: the combined effects of multiple drugs or enzyme inhibitors. *Adv Enzyme Regul* **1984**, *22*, 27-55, doi:10.1016/0065-2571(84)90007-4.
2. Chou, T.C. Theoretical basis, experimental design, and computerized simulation of synergism and antagonism in drug combination studies. *Pharmacol Rev* **2006**, *58*, 621-681, doi:10.1124/pr.58.3.10.

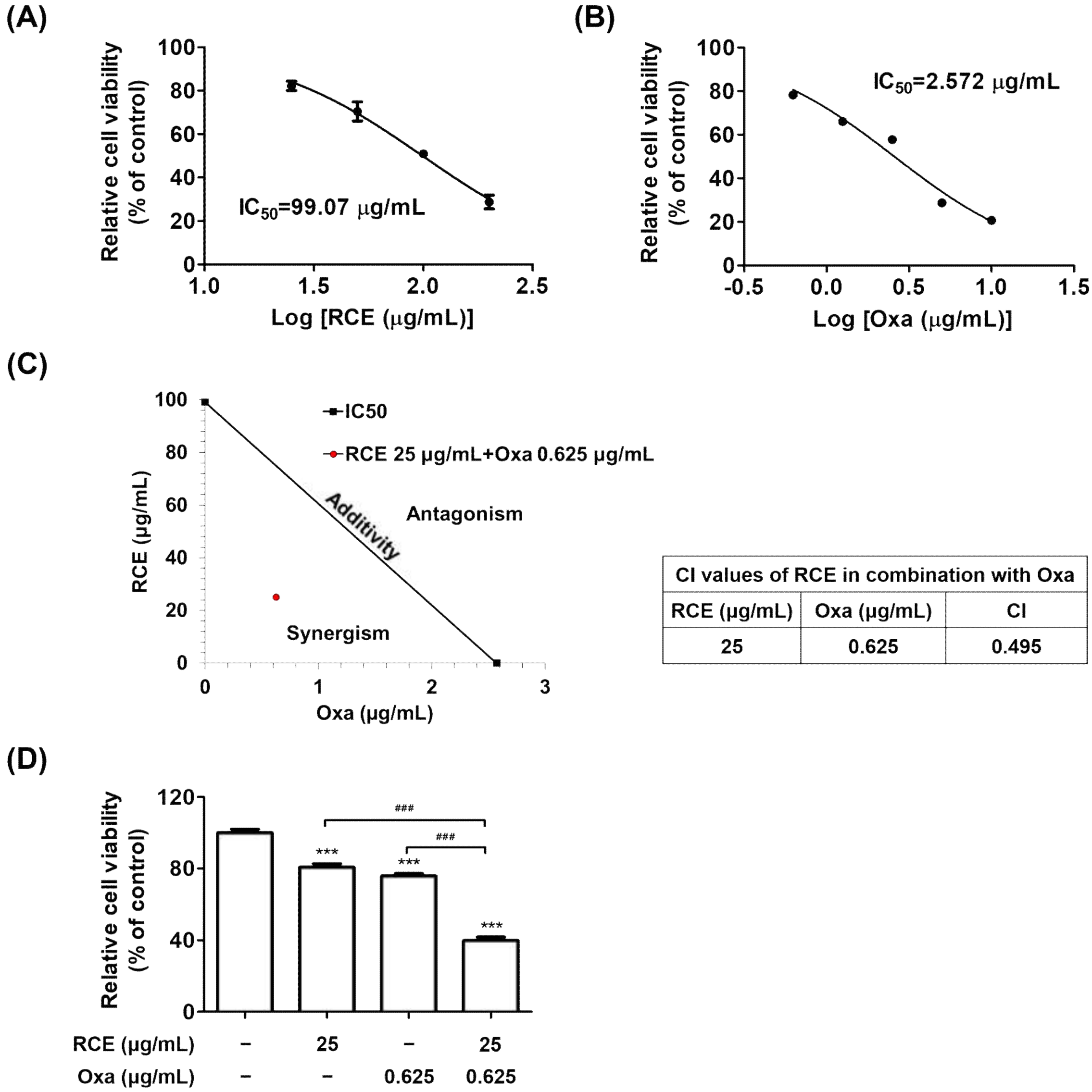

CI values of RCE in combination with Oxa		
RCE (μg/mL)	Oxa (μg/mL)	CI
25	0.625	0.495

Figure S1. Synergistic effects of RCE plus Oxa on the co-culture of hPD-1 tumor-infiltrating $CD3^+CD8^+$ T cells and hPD-L1 MC38 cells. **(A, B)** The IC_{50} value of hPD-L1 MC38 cells co-cultured with tumor-infiltrating $CD3^+CD8^+$ T cells was determined by the CCK assay treated with various concentrations of RCE (0–200 μg/mL) (A) or Oxa (0–10 μg/mL) **(B)** for 72 h. **(C)** Isobologram analysis. The diagonal lines indicate additivity while the red symbol indicates the dose requirement to achieve 50% cell viability inhibition. Data points below the line of additivity indicate synergism while data points above indicate antagonism. The table presents the CI values of RCE plus Oxa on the co-culture of hPD-1 tumor-infiltrating $CD3^+CD8^+$ T cells and hPD-L1 MC38 cells. **(D)** The viability of hPD-L1 MC38 cells co-cultured with tumor-infiltrating $CD3^+CD8^+$ T cells was evaluated using the CCK assay in the RCE alone (25 μg/mL), Oxa alone (0.625 μg/mL), and RCE plus Oxa groups. The results are presented as the mean ± SD. $^{***}p < 0.001$ compared with the control, $^{###}p < 0.001$ compared with the individual group.
