# Supplementary figures and images for: Augmented Antitumor Effect of Unripe Rubus coreanus Miquel Combined with Oxaliplatin in a Humanized PD-1/PD-L1 Knock-In Colorectal Cancer Mouse Model
Source: Cells. 2022 Sep 14;11(18):2876. doi: 10.3390/cells11182876 (PMC9496898; doi:10.3390/cells11182876)

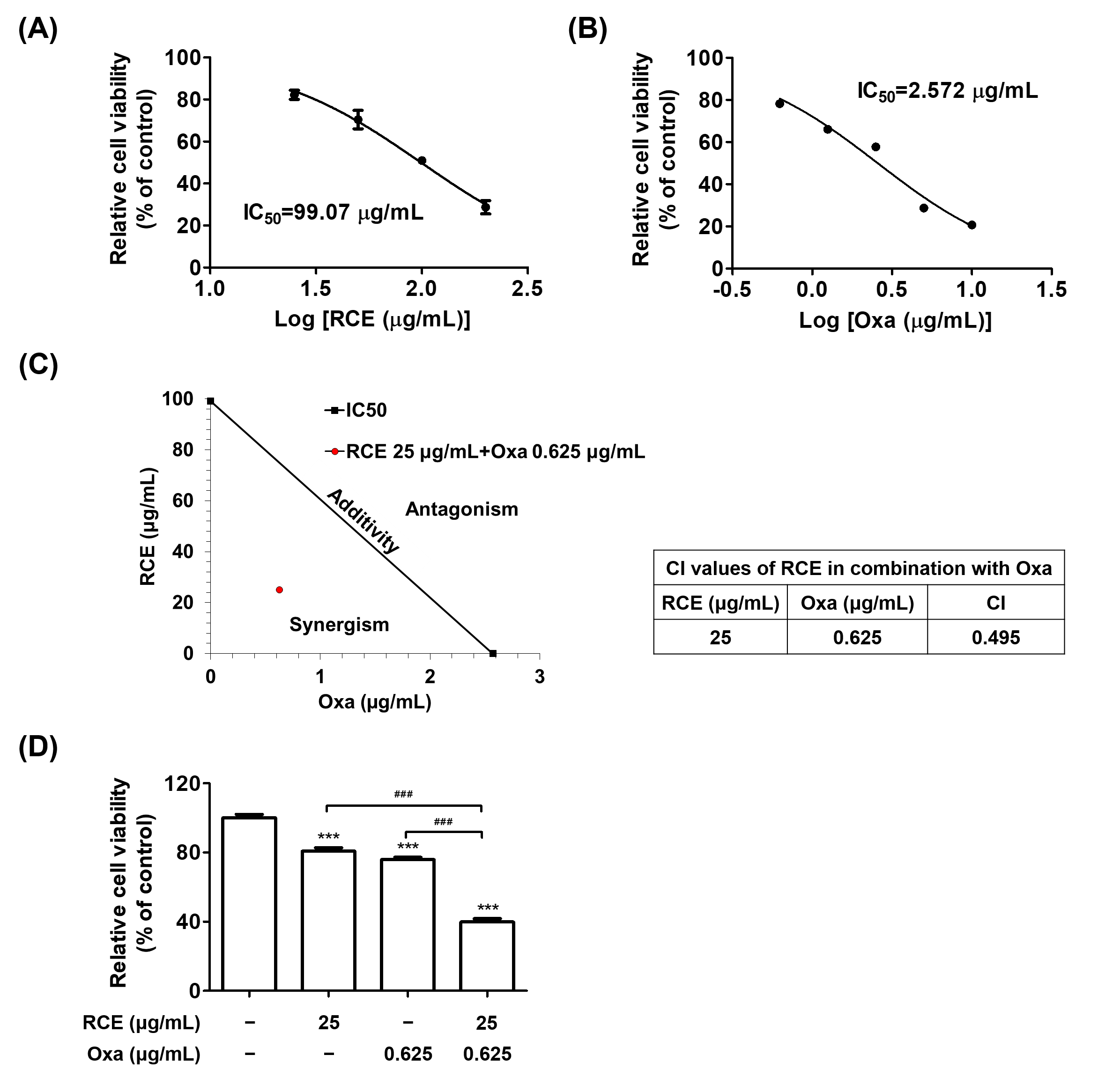

Supplement: Supplementary file 1 [file cells-11-02876-s001.zip › Figure S1.tif]
